# Co-overexpression of Bag-1 and heat shock protein 70 in human epidermal squamous cell carcinoma: Bag-1-mediated resistance to 5-fluorouracil-induced apoptosis

**DOI:** 10.1038/bjc.2011.111

**Published:** 2011-04-26

**Authors:** J Wood, M Pring, J W Eveson, N Price, C M Proby, A Hague

**Affiliations:** 1 School of Oral and Dental Science, University of Bristol, Lower Maudlin Street, Bristol BS1 2LY, UK; 2 Skin Tumour Laboratory, Wellcome Centre for Molecular Medicine, PO Box 11, Ninewells Hospital and Medical School, University of Dundee, Dundee DD1 9SY, UK

**Keywords:** Bag-1, heat shock protein-70, skin, apoptosis, immunohistochemistry, 5-fluorouracil

## Abstract

**Background:**

The aim was to determine whether Bcl-2-associated athanogene-1 (Bag-1) and/or its binding protein heat shock protein-70 (Hsp70) exhibit deregulated expression in epidermal squamous cell carcinoma (SCC) and whether Bag-1 confers apoptosis resistance.

**Method:**

Immunohistochemistry for Bag-1 and Hsp70 was performed on 60 epidermal SCC and 10 normal skin samples. The epidermal SCC cell line SCC-13 was treated with 5-fluorouracil (5-FU) after Bag-1 knockdown to determine whether high Bag-1 levels contribute to growth and/or apoptosis resistance.

**Results:**

Normal epithelium expressed primarily nuclear Bag-1. Most tumours showed reduced nuclear Bag-1 staining, but a subset exhibited strong Bag-1 staining, with cytoplasmic Bag-1 staining intensity correlating with cytoplasmic Hsp70 staining intensity (*r*_s_=0.462; *P*<0.001) and less differentiation (*P*<0.001). Bag-1 knockdown resulted in markedly reduced SCC-13 cell yield, increased spontaneous apoptosis and enhanced sensitivity to 5-FU-induced apoptosis. Apoptosis induced by 5-FU in the Bag-1-knockdown cells was significantly greater than the additive apoptotic effect of 5-FU or Bag-1 knockdown alone.

**Conclusions:**

Overexpression of Bag-1 and Hsp70 in poorly differentiated SCC may confer both enhanced tumour cell growth and apoptosis resistance. Bag-1 may contribute to the resistance of more advanced epidermal SCC to chemotherapy.

Bcl-2-associated athanogene-1 (Bag-1) expression is deregulated in a variety of human tumours, including cancers of the breast, lung, colon, oesophagus, larynx, oral cavity and tongue (reviewed by Wood *et al*, 2009). The Bag-1 protein was first identified because of the interaction with Bcl-2, and was shown to enhance Bcl-2-mediated cell survival (Takayama *et al*, 1995). Bag-1 interacts with various cellular proteins in addition to Bcl-2, including Raf-1 and B-Raf (Wang *et al*, 1996; Götz *et al*, 2005), retinoblastoma protein (Arhel *et al*, 2003), GADD34 (Hung *et al*, 2003), growth factor receptors (Bardelli *et al*, 1996), nuclear receptors (Zeiner and Gehring, 1995), heat shock proteins (Hsps) Hsc70 and Hsp70 (Höhfeld and Jentsch, 1997; Zeiner *et al*, 1997), the proteasome (Lüders *et al*, 2000) and E3 ligases, Siah-1 (Matsuzawa *et al*, 1998) and CHIP (Alberti *et al*, 2002).

Bag-1 expression is deregulated in oral squamous cell carcinoma (SCC), with increased expression associated with progression, metastasis and poor prognosis in early stage tumours (Shindoh *et al*, 2000) and increased cytoplasmic Bag-1 in lymph node metastases (Hague *et al*, 2002). Bag-1 has been implicated as a prognostic marker in oral SCC, showing correlation with overexpression of Bcl-2 (Xie *et al*, 2004) and Hsp70 (Weber *et al*, 2007). Three major Bag-1 isoforms are expressed in human cells, Bag-1S, Bag-1M and Bag-1L, with molecular sizes of 36, 46 and 50 kDa, respectively. Some tissues and tumour cells express a fourth Bag-1 isoform, 29 kDa in size (Townsend *et al*, 2003a). Although Bag-1L is predominantly nuclear, Bag-1M and Bag-1S partition between cytoplasm and nucleus (Yang *et al*, 1998). Relocalisation to the nucleus at 2 h after heat shock has been shown for Bag-1M in HeLa cells (Zeiner *et al*, 1999) and Bag-1S in MCF-7 breast cancer cells (Townsend *et al*, 2003b). By contrast, in colorectal tumour cells Bag-1M exited the nucleus 24 h after *γ*-irradiation (Barnes *et al*, 2005). Moreover, overexpression of nuclear Bag-1L conferred protection against apoptosis in response to *γ*-irradiation and the vitamin D analogue, EB1089 (Barnes *et al*, 2005). Together, these findings indicate a nuclear site of action for the anti-apoptotic function of Bag-1. Nuclear anti-apoptotic functions can be mediated by Bag-1 interaction with Hsp70 (Townsend *et al*, 2003b).

Hsp70/Hsc70 interactions with Bag-1 may also be required for an anti-apoptotic function in the cytoplasm. In cardiac myocytes, Bag-1S and Bag-1M, but not Bag-1L, were protective against apoptosis following simulated ischemia–reperfusion injury (Townsend *et al*, 2004). Bag-1S with point mutations in the Hsc70/Hsp70 binding domain was unable to confer apoptosis protection. The requirement for a cytoplasmic localisation for the anti-apoptotic effect of Bag-1 was demonstrated by the overexpression of Bag-1S tagged with a nuclear localisation sequence (localised entirely in the nucleus) which failed to protect against apoptosis (Townsend *et al*, 2004). Bag-1 protects against apoptosis induced by various other stimuli including Fas signalling (Takayama *et al*, 1995), growth factor withdrawal (Clevenger *et al*, 1997), hypoxia (Ishioka *et al*, 2007) and cytotoxic drugs (Chen *et al*, 2002). Studies indicating a role for Hsp70 binding as a mechanism for Bag-1 anti-apoptotic function suggest that co-expression of these proteins may facilitate increased resistance to apoptosis in cancer, with potential implications for treatment, such as acquisition of resistance to chemotherapeutic drugs.

Non-melanoma skin cancer has a rapidly increasing incidence, the main aetiological agent being ultraviolet B (UVB) radiation, a complete carcinogen (Assefa *et al*, 2005). Non-melanoma skin cancer is the most common human cancer, with ∼100 000 new cases diagnosed per year in the United Kingdom, of which around 20% are SCC (source: Cancer Research UK). In particular, patients taking immunosuppressive drugs have a 100-fold higher risk of developing cutaneous SCC, which is specific to sun exposed skin areas (Lindelöf *et al*, 2000). Sun exposure-induced skin cancers frequently show ‘UV-signature’ CC to TT base transition mutations in the *p53* gene (Boukamp, 2005). p53 gain-of-function mutants have previously been shown to upregulate Bag-1 expression (Yang *et al*, 1999) and a correlation between Bag-1 and p53 expression has been shown in breast cancer (Tang *et al*, 2004). Surgery is successful in treating most SCCs, but radiotherapy and chemotherapy are also used. Chemotherapy creams containing 5-fluorouracil (5-FU) are used to treat actinic keratosis, a pre-malignant lesion, and Bowen's disease (SCC *in situ*; Moore, 2009; Micali *et al*, 2010). 5-FU is an anti-metabolite, which is converted to fluorouridine monophosphate, inhibiting thymidylate synthase and hence interfering with DNA synthesis, as well as being further metabolised to uridine analogues that are incorporated into RNA and DNA. The resultant cellular responses are growth inhibition and cell death. As actinic keratoses occur on sun-exposed skin and patients typically have multiple lesions, often on the face, 5-FU ointments are favoured over surgery to treat the field. They are also used on digits to preserve mobility (Moore, 2009).

Although in oral epithelium, the spontaneous frequency of apoptosis is very low (Hague *et al*, 2004), in epidermis there is a very high rate of apoptosis at sun-exposed sites and overactive survival pathways may contribute to tumourigenesis in the skin (Lippens *et al*, 2009). However, there are no currently published studies investigating the expression of the anti-apoptotic proteins Bag-1 or Hsp70 in epidermal SCC. Here we used an immunohistochemical study of human epidermal SCC to determine whether Bag-1 and/or Hsp70 expression was altered during squamous cell carcinogenesis of the skin, and whether Bag-1 expression was related to Hsp70 or p53 staining. There are no reports of whether Bag-1 protects against apoptosis in epidermal SCC and whether Bag-1 might affect response to therapy. Therefore, we used 5-FU as an apoptotic stimulus and a Bag-1 knockdown approach to determine whether the high levels of Bag-1 expressed in the epidermal SCC cell line SCC-13 contribute to apoptosis resistance.

## Materials and methods

### Tissues, antibodies and siRNA

Formalin-fixed, paraffin-embedded tissue from epidermal SCC (60) and normal epidermis overlying an epidermoid cyst (10) was used (local ethics committee approval: 07/H0104/72). Patient and SCC characteristics are summarised in Table 1. Antibodies used were rabbit polyclonal antibody (TB3) raised against a Bag-1-GST fusion protein (Packham *et al*, 1997; kind gift from G Packham, University of Southampton), mouse monoclonal anti-Hsp70 antibody (SMC-100, clone C92F3A-5, StressMarq Biosciences Inc., York, UK) and mouse monoclonal anti-human p53 antibody (BP53-12, Novocastra Laboratories Ltd, Newcastle, UK). For Bag-1-knockdown experiments, a Silencer control siRNA (ID #4635) and three siRNAs specific for Bag-1 mRNA were used: ID #5063 targets exon 2, ID #5156 targets exons 2 and 3 and ID #147325 targets exon 4 (Ambion, Applied Biosciences Europe, Warrington, UK).

### Immunohistochemistry

Tissue sections (2 *μ*m) were mounted on poly-lysine coated slides and incubated overnight at 37°C, deparaffinised in xylene, rehydrated in a decreasing ethanol series, then rinsed in distilled water. Antigen retrieval was carried out by microwaving (25 min at 800 W in 1 l of citrate buffer, pH 6.0). Endogenous peroxidase activity was blocked for 15 min in 3% (v/v) hydrogen peroxide in distilled water. Slides were incubated with anti-Bag-1, Hsp70 or p53 primary antibody (1 : 1000, 1 : 50 and 1 : 50, respectively, in phosphate-buffered saline (PBS), pH 7.2, containing 1% normal goat serum (NGS)) at room temperature for 1 h. Antibody binding was detected using Super Sensitive Link-label IHC detection system (BioGenex, San Ramon, CA, USA). Diaminobenzidine peroxidase substrate (Dako North America Inc., Carpinteria, CA, USA) was applied for 6 min, followed by washing in distilled water and a 40 s Mayer's haematoxylin counterstain. Sections were dehydrated using ethanol, cleared in xylene and mounted using DPX mountant (Fisher Scientific Ltd, Loughborough, UK).

Negative controls (1% NGS alone) were included in each staining run. Specificity of Bag-1 recognition by TB3 antibody was confirmed using antiserum depleted of Bag-1 antibody by pre-adsorption with GST–Bag-1 protein. In addition, the HaCaT skin keratinocyte cell line was transfected at 30% confluence with a trio of siRNAs specific for the Bag-1 mRNA (10 nM each; 30 nM total) or with Silencer control siRNA (30 nM) in Opti-MEM medium (Invitrogen, Carlsbad, CA, USA) with Oligofectamine (Invitrogen). At 72 h post transfection, cells were fixed using formal saline, paraffin-embedded and stained with TB3 antibody.

### Cell lines and cell culture

#### Normal epidermal keratinocytes

Normal epidermal keratinocytes (derived from human breast skin) were cultured on 3 : 1 Dulbecco's modified Eagles medium (DMEM): Ham's F12 (Invitrogen, Paisley, UK) with 10% fetal bovine serum (FBS; Autogen Bioclear, Wiltshire, UK), 0.5 *μ*g ml^−1^ hydrocortisone, 10^−10^ M cholera toxin, 5 *μ*g ml^−1^ transferrin, 2 × 10^−11^ M liothyronine (all from Sigma-Aldrich, Gillingham, Dorset, UK), 5 *μ*g ml^−1^ insulin and 10 ng ml^−1^ human recombinant EGF (Promega, Southampton, Hampshire, UK) in the presence of mitomycin C-treated Swiss 3T3 feeders.

#### HaCaT cells

The spontaneously immortalised HaCaT epidermal keratinocyte cell line, which harbours UV-typical *p53* gene mutation (Boukamp *et al*, 1988) was used for Bag-1-knockdown experiments to validate the specificity of the TB3 Bag-1 antibody. HaCaT cells were cultured on 1 : 1 DMEM:Ham's F12 (Biowhittaker Lonza, Verviers, Belgium) with 10% FBS and 0.5 *μ*g ml^−1^ hydrocortisone.

#### Squamous cell carcinoma cell lines

The PM1, PM3, MET2 and MET4 cell lines were all derived from a 45-year-old male renal transplant recipient (Proby *et al*, 2000). These cell lines were extensively tested for human papillomavirus and were shown to be negative using a PCR-based method designed to detect all known cutaneous, epidermodysplasia verruciformis and mucosal HPV types with a high sensitivity equivalent to 0.05 HPV genome copies per cell (Proby *et al*, 2000). The MET2 and MET4 cell lines have a set of common translocations, suggesting common origin, and have wild-type *ras* genes (Popp *et al*, 2000). PM1 and PM3 were derived from dysplastic adult forehead skin and are non-tumourigenic, whereas MET2 was derived from a recurrence of SCC on the back of the hand, and MET4 from metastatic SCC within the left axillary lymph nodes (Proby *et al*, 2000). PCR for exons 5–8 of the *p53* gene did not reveal any mutations (Popp *et al* 2000), however, MET2 has recently been found to have a homozygous *p53* mutation resulting in amino acid change V216M, which may have resulted from expansion of a mutant *p53* cell population *in vitro* (Andrew South, University of Dundee, personal communication). SCC-12 and SCC-13 are derived from facial epidermal SCCs. SCC-12 was from a 60-year-old male renal transplant patient and SCC-13 was from a 56-year-old female patient who had received a series of radiation treatments for the tumour several years before its surgical removal (Rheinwald and Beckett, 1981). SCC-12 has a V216G and SCC-13 has a Q258K p53 mutation (Burns *et al*, 1993). BICR19 was derived from an SCC of the ear of a 69-year-old male and has a *p53* mutation (exon 10 deletion) and expresses in addition a normal *p53* allele (Burns *et al*, 1993; Edington *et al*, 1995; Ken Parkinson, Queen Mary, University of London; Keith Hunter, University of Sheffield, personal communication). The PM1, PM3, MET2 and MET4 were cultured on 3 : 1 DMEM: Ham's F12 with 10% FBS, 0.5 *μ*g ml^−1^ hydrocortisone, 10^−10^ M cholera toxin, 5 *μ*g ml^−1^ transferrin, 2 × 10^−11^ M liothyronine, 5 *μ*g ml^−1^ insulin and 10 ng ml^−1^ human recombinant EGF in the presence of mitomycin C-treated Swiss 3T3 feeders. The SCC-12, SCC-13 and BICR19 cell lines were cultured on DMEM with 10% FBS and 0.5 *μ*g ml^−1^ hydrocortisone. To compare the levels of Bag-1, all cell lines were transferred onto 3T3 conditioned dishes in 3T3 conditioned medium consisting of 3 : 1 DMEM: Ham's F12 with 10% FBS, with supplements added after conditioning: 0.5 *μ*g ml^−1^ hydrocortisone, 10^−10^ M cholera toxin, 5 *μ*g ml^−1^ transferrin, 2 × 10^−11^ M liothyronine and 5 *μ*g ml^−1^ insulin. Cells were cultured until ∼50% confluent (typically 3–4 days) before harvest for western blotting.

### Bag-1 knockdown and 5-FU treatment in the SCC-13 cell line

The SCC-13 cell line was treated with 5-FU in DMEM with 10% FBS and 0.5 *μ*g ml^−1^ hydrocortisone. For Bag-1 knockdown, the SCC-13 cell line was transfected with a trio of siRNAs specific for the Bag-1 mRNA (3.33 nM each; 10 nM total) or with 10 nM Silencer control siRNA at 30% confluence, as described above. At 48 h after Bag-1 knockdown, cells were treated with 20 *μ*M 5-FU (Sigma-Aldrich) dissolved in DMSO, or with DMSO only, for 48 h.

### Measurement of 5-FU-induced apoptosis

Methods for measuring apoptosis based on collection of dead cells shed into the culture medium have been described previously (Hague *et al*, 1993; Díaz *et al*, 2000). In two experiments, western blotting samples were made from pooled adherent and floating cells. In the third experiment, cells were used to confirm apoptotic morphology of the floating cells by staining live cells for 10 min with a mixture of acridine orange and ethidium bromide in PBS, each at 5 *μ*g ml^−1^.

### Western blotting

Cells were lysed on ice using RIPA buffer (0.15 M NaCl, 1% (v/v) Nonidet P-40 (NP-40), 0.5% (w/v) sodium deoxycholate, 0.1% (w/v) SDS, 50 mM Tris-HCl (pH 8)) containing protease inhibitors (Cocktail set III, Calbiochem, Nottingham, UK) and phosphatase inhibitors (PhosSTOP, Roche, Welwyn Garden City, Hertfordshire, UK). Lysates were clarified by centrifugation at 15 800 *g* for 20 min at 4°C. Protein concentration was measured using the BioRad DC Protein Assay Kit (BioRad Laboratories, Hercules, CA, USA). A volume containing 60 *μ*g protein was loaded onto 7.5% acrylamide gels for SDS–PAGE. Proteins were transferred to Immobilon-P polyvinylidene difluoride membrane (Millipore, Consett, County Durham, UK) by semi-dry electroblotting. Membranes were blocked for 1 h in Milk block buffer (1% 10 mM Tris/HCl (pH7.4), 150 mM NaCl, 4% fat free milk powder), and were then incubated with primary antibody on a rotator at 4°C overnight. Incubation with *α*-tubulin was carried out at room temperature for 30 min. Antibodies used were mouse anti-Bag-1 (G3E2, hybridoma culture supernatant, gift from G Packham, University of Southampton, 1 : 100), mouse anti-human p53 (clone 1801, hybridoma culture supernatant, gift of Ann Williams, University of Bristol, 1 : 100), mouse anti-Hsp70 (SMC-100, StressMarq Biosciences Inc., 1 : 10 000), mouse anti-*α*-tubulin (T9026, clone DM1A, Sigma-Aldrich, 1 : 10 000) and mouse anti-poly-(ADP-ribose) polymerase (PARP; AM30, Calbiochem, 1 : 100). After a series of washes in Milk block buffer and Tween buffer (1% 10 mM Tris/HCl (pH7.4), 150 mM NaCl and 0.2% Tween-20) membranes were incubated with HRP-conjugated sheep anti-mouse secondary antibody (Sigma-Aldrich, 1 : 1000) for 1 h followed by further washes and distilled water rinse. Peroxidase activity was measured using ECL (Amersham Biosciences, Buckinghamshire, UK) and imaged using Kodak MXB film (Xograph Imaging Systems, Gloucestershire, UK).

### Scoring and data analysis

Two independent investigators (MP and JWE) scored the normal and tumour sections as no staining (0), weak (+), moderate (++) or strong (+++) for cytoplasmic and nuclear staining separately. The intensity of the majority of the section was recorded, as was percentage positivity. In cases of slight discrepancy, slides were re-examined by the original scorers and an agreed score was assigned. Statistical analysis was performed with SPSS software (IBM Corporation, Somers, NY, USA), using Kruskall–Wallis, Mann–Whitney and Spearman's *ρ* correlation tests. Tumour and patient characteristics were grouped for statistical analysis as shown in Table 1.

## Results

### Bag-1 expression in normal human skin and epidermal SCC

**Figure 1:**
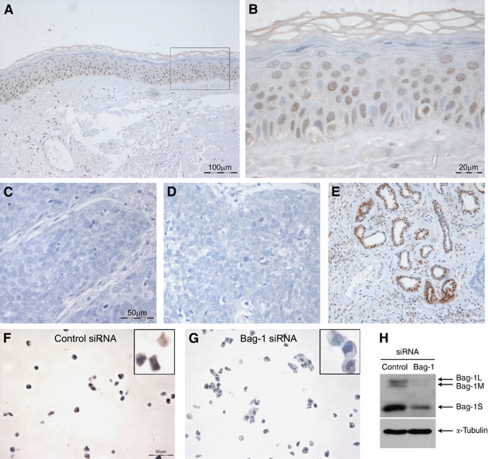
Bag-1 immunohistochemistry of normal human skin. (**A**) Bag-1 expression in normal human skin. (**B**) A higher power image of boxed region in **A**, illustrating predominantly nuclear localisation of Bag-1. Negative controls were: (**C**) negative control tumour section without primary antibody; corresponding Bag-1 stained tumour showed 80% positive cells (assigned staining intensity scores of nuclear +++, cytoplasmic ++) and (**D**) rabbit pre-immune serum; corresponding Bag-1 stained tumour showed 80% positive cells (assigned staining intensity scores of nuclear +++, cytoplasmic ++). (**E**) Strong Bag-1 staining in sweat glands was used as an internal positive control to ensure consistent staining. (**F**, **G** and **H**) Validation of Bag-1 TB3 antibody using small interfering RNA (siRNA) knockdown in the HaCaT keratinocyte cell line. HaCaT cells were transfected with Silencer control siRNA (**F**) or Bag-1 siRNA (**G**). (**H**) Western blot showing reduced expression of Bag-1 protein in HaCaT cells transfected with Bag-1 siRNA compared with Silencer control siRNA. Images **A** and **E** were photographed using a × 10 objective (scale bar shown in **A**), images **C**, **D**, **F** and **G** were photographed using a × 20 objective (scale bars shown in **C** and **F**), and image **B** using a × 40 objective (scale bar shown).

Bag-1 expression in the epidermis of normal human skin was found to be primarily nuclear, with expression in the basal and suprabasal layers and reduced expression towards the upper epithelial layers, with loss of expression in the cornified layer (Figures 1A and B). Some scattered nuclei were negative for Bag-1 staining. Negative controls with PBS containing normal goat serum only or pre-immune rabbit serum showed no staining (Figures 1C and D, respectively), whereas sweat gland ducts were always strongly stained and served as a useful internal positive control (Figure 1E). Lymphocytes, sebaceous glands and the base of the hair follicles were also consistently strongly stained (data not shown). As a further control, Bag-1 staining of HaCaT cells transfected with either Bag-1 siRNA or Silencer control siRNA were compared after paraffin embedding and sectioning; Bag-1 staining was reduced in the HaCaT cells after Bag-1 knockdown (Figure 1G) compared with Silencer control-treated cells (Figure 1F). The extent to which Bag-1 expression was reduced was investigated by western blotting (Figure 1H). In HaCaT cells Bag-1S is the predominant isoform, but Bag-1M and Bag-1L are also expressed. All three Bag-1 protein isoforms are reduced by siRNA knockdown.

**Figure 2:**
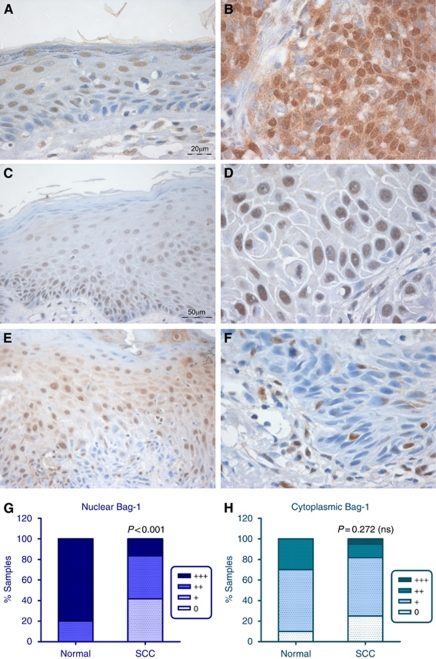
Expression of Bag-1 in epidermal SCC and adjacent epithelium. (**A**, **C** and **E**) Bag-1 expression in epithelium adjacent to SCC; note the altered pattern of Bag-1 expression in adjacent epithelium, compared with the normal skin expression pattern shown in Figures 1A and B. (**B**) Moderate to poorly differentiated SCC showing increased cytoplasmic and nuclear Bag-1 expression relative to adjacent epithelium (**A**). (**D**) Well-differentiated SCC showing reduced cytoplasmic and increased nuclear Bag-1 expression, compared with adjacent epithelium (**C**). (**F**) Well-differentiated SCC showing reduced Bag-1 expression relative to adjacent dysplastic epithelium (**E**). Images **C** and **E** were photographed using a × 20 objective (scale bar shown in **C**), and images **A**, **B**, **D** and **F** were photographed using a × 40 objective (scale bar shown in **A**). (**G**) Comparison of intensity of Bag-1 nuclear staining in normal epidermis compared with the panel of SCCs. (**H**) Comparison of Bag-1 cytoplasmic staining in normal epidermis compared with the panel of SCCs. Statistical analysis was performed using a Mann–Whitney test.

Within the tissue samples of SCC, epithelium adjacent to the tumours showed a slightly different pattern of Bag-1 expression compared with normal epidermis, with reduced basal Bag-1 staining (compare Figure 2A with Figure 1B). The adjacent epithelium was in some cases histologically normal (Figure 2A), in some cases hyperplastic (Figure 2C) and in some cases dysplastic (Figure 2E). The intensity of Bag-1 staining was variable (compare Figures 2A, C and E). Some of the adjacent epithelia showed evidence of sun damage, including thickened dermal tissue and deeply penetrating rete pegs (data not shown). Tumours varied in expression of Bag-1, although some showed pronounced nuclear and cytoplasmic staining greater than that of the corresponding adjacent epithelium (compare Figures 2A and B), others exhibited primarily nuclear staining of similar intensity to suprabasal cells (compare Figures 2C and D) and some tumours showed weak or no Bag-1 staining compared with adjacent, non-invasive epithelium (compare Figures 2E and F). The cytoplasmic scores for Bag-1 intensity were not significantly different between the normal tissue samples and the SCC samples (*P*=0.272; Figure 2H), whereas there was a highly significant difference in nuclear Bag-1 staining between the normal samples and SCC samples (*P*<0.001; Figure 2G). The overall picture is of reduced intensity of nuclear Bag-1 staining in the tumours compared with normal skin epithelium. However, a subset of tumours showed moderate to strong Bag-1 staining. The statistical analyses performed and *P*-values obtained in this study are summarised in Table 2.

### Hsp70 staining of normal skin and epidermal SCC

**Figure 3:**
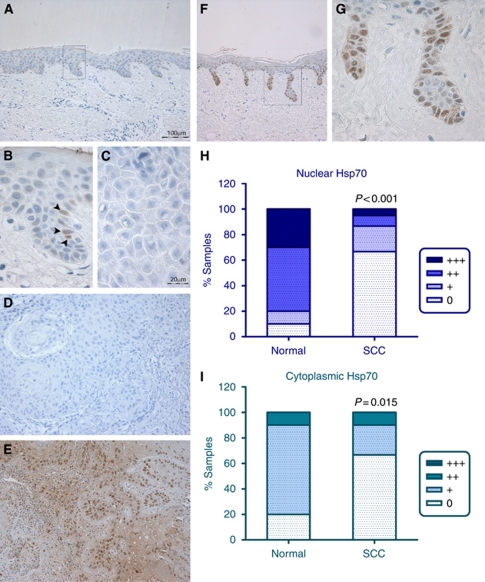
Hsp70 immunohistochemistry of normal human skin, epidermal SCC and adjacent epithelium. (**A**) Normal epidermal epithelium is predominantly negative for Hsp70 staining, with occasional Hsp70-positive cells, located within the basal layer. A higher power image of the boxed area in **A** is shown in **B**, to highlight the presence of occasional Hsp70-positive cells (arrowheads). (**C**) Negative control (normal goat serum in PBS) tumour section without primary antibody; the corresponding Hsp70 stained tumour showed positive staining in 30% of the cells (assigned staining intensity scores of nuclear ++, cytoplasmic +). (**D**) Tumour negative for Hsp70 staining. (**E**) Hsp70-positive staining human epidermal SCC. (**F** and **G**) Deep peg-like rete ridges stained strongly positive for Hsp70 in sun-damaged epidermis adjacent to SCC; boxed area in **F** is shown at higher magnification in **G**. (**H**) Intensity of Hsp70 nuclear staining in normal epithelium and in the panel of SCCs. (**I**) Intensity of Hsp70 cytoplasmic staining in normal epithelium and in the panel of SCCs. Images **A**, **F**, **D** and **E** were photographed using a × 10 objective (scale bar shown in **A**). Images **B**, **C** and **G** were photographed using a × 40 objective (scale bar shown in **C**). Statistical analysis was performed using a Mann–Whitney test. 0=undetectable Hsp70 staining; +=weak; ++=moderate; +++=strong Hsp70 staining.

Normal epidermis was generally negative for Hsp70 staining at the antibody concentration used, with occasional Hsp70-positive cells located within the basal layer (Figures 3A and B). Indeed, the majority of SCCs were negative for Hsp70 staining (40/60 tumours; 66.7% Figure 3D), and there was strong evidence for reduced Hsp70 staining intensity in the tumours compared with the normal epidermal samples (*P*<0.001 and *P*=0.015 for nuclear and cytoplasmic Hsp70, respectively). The remaining 33.3% of tumours exhibited weak to moderate cytoplasmic Hsp70 staining and weak to strong nuclear staining (Figures 3H and I). Hence, where positive, tumours exhibited both nuclear and cytoplasmic staining (Figure 3E). Interestingly, the sun-damaged epithelium adjacent to some of the tumours showed pronounced Hsp70 staining. In particular, the deep rete pegs contained strongly Hsp70-positive basal cells (Figures 3F and G).

### Bag-1 staining and Hsp70 staining are inversely associated with differentiation of the tumour

**Figure 4:**
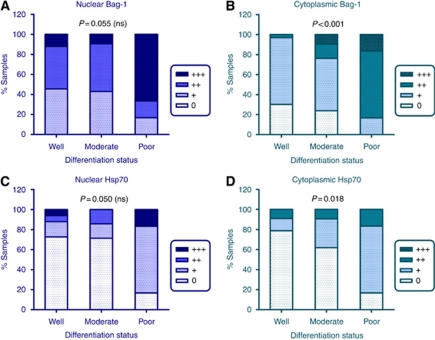
Expression of Bag-1 and Hsp70 in human SCC in relation to differentiation of the stained section. (**A**) Intensity of Bag-1 nuclear staining in well, moderately and poorly differentiated tumours. (**B**) Intensity of cytoplasmic Bag-1 staining in well, moderately and poorly differentiated tumours. (**C**) Intensity of nuclear Hsp70 staining in well, moderately and poorly differentiated tumours. (**D**) Intensity of cytoplasmic Hsp70 staining in well, moderately and poorly differentiated tumours. Statistical analysis was performed using a Kruskal–Wallis test. The numbers of tumours in each differentiation category were as follows: well (33), moderate (21), poor (6). ns, nonsignificant. 0=undetectable staining; +=weak; ++=moderate; +++=strong staining.

Overall, the tumours showed no change in cytoplasmic Bag-1 staining intensity compared with normal epidermal epithelium (Figure 2H). However, examining the series of tumours further, there was an inverse association between SCC differentiation and intensity of cytoplasmic Bag-1 staining in the sections (*P*<0.001). These data are illustrated in Figure 4, which shows that well differentiated tumours tended to have weak or no cytoplasmic Bag-1 staining, with the intensity of cytoplasmic staining increasing through moderate to poorly differentiated SCC (Figure 4B). The relationship between differentiation and nuclear Bag-1 staining was less pronounced (*P*=0.055). Overall, tumours showed reduced levels of nuclear Bag-1 compared with normal epidermis (Figure 2G). Interestingly, although well- and moderately differentiated tumours show reduced nuclear staining compared with normal epidermis (compare Figure 4A with Figure 2G), the poorly differentiated tumours tended to show nuclear Bag-1 expression patterns more similar to those of normal epidermis. This may reflect an increase in both nuclear and cytoplasmic Bag-1 expression in the poorly differentiated tumours.

The percentage of Bag-1 positive cells in the tumour also showed an association with differentiation (*P*=0.007), indicating a link between Bag-1 protein and reduced differentiation status. Interestingly, expression of Hsp70 in the tumours also appeared to show an inverse association with differentiation (Figures 4C, D). Cytoplasmic Hsp70 staining was inversely associated with tumour differentiation (*P*=0.018; Figure 4D), with nuclear Hsp70 staining just nonsignificant (*P*=0.050; Figure 4C). The percentage of cells staining positively for Hsp70 also showed an inverse relationship with the extent of differentiation of the tumour (*P*=0.011).

### Co-expression of Bag-1 and Hsp70 in epidermal SCC

**Figure 5:**
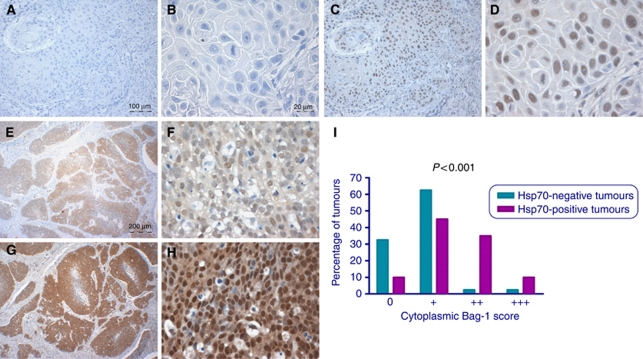
Correlation between Bag-1 and Hsp70 expression. (**A** and **B**) Epidermal SCC negative for Hsp70 staining. (**C** and **D**) Corresponding areas of the same tumour stained for Bag-1 showing weak cytoplasmic Bag-1 expression and moderate nuclear staining. (**E** and **F**) Epidermal SCC showing a moderate level of cytoplasmic and nuclear Hsp70 staining. (**G** and **H**) Corresponding areas of the same tumour shown in **E** and **F** stained for Bag-1, showing strong cytoplasmic and nuclear Bag-1 staining. Images **E** and **G** were photographed using a × 4 objective (scale bar shown in **E**), images **A** and **C** were photographed using a × 10 objective (scale bar shown in **A**) and images **B**, **D**, **F** and **H** using a × 40 objective (scale bar shown in **B**). (**I**) Relationship between Hsp70 positivity and intensity of Bag-1 staining in SCCs. Statistical analysis was performed using a Mann–Whitney test. 0=undetectable Bag-1 staining; +=weak; ++=moderate; +++=strong cytoplasmic Bag-1 staining scores.

In serial sections, areas of tumour positive for cytoplasmic Hsp70 tended to exhibit strong cytoplasmic Bag-1 staining (Figure 5) and there was a clear association between intensity of Hsp70 and Bag-1 staining, with a correlation between cytoplasmic Bag-1 and Hsp70 staining intensity scores (*P*<0.001; correlation coefficient=0.462). There was also a weak correlation between the percentage of cells that stained positively for Hsp70 in the tumours and the percentage that were positive for Bag-1 (*P*=0.027; correlation coefficient=0.286). However, there was no association between nuclear staining intensities for these two proteins (*P*=0.067).

### Association between p53-positive staining and nuclear Bag-1 staining intensity

**Figure 6:**
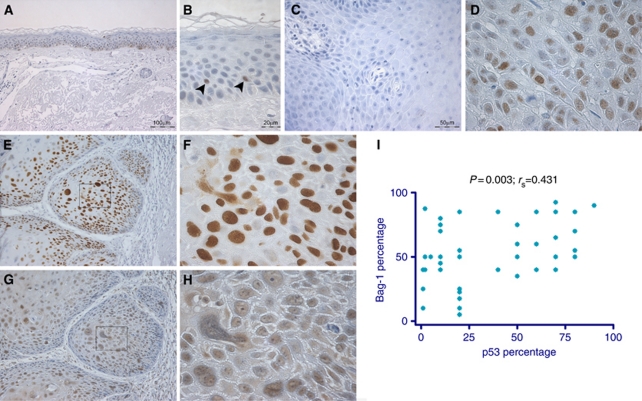
p53 staining patterns in normal epidermis and SCC and association with Bag-1 expression. (**A** and **B**) p53 expression in normal epidermis. Normal skin is predominantly negative for p53, with some occasional p53-positive cells. A small number of p53-positive cells can be seen (arrowheads in **B**). (**C**) Negative control tumour section without primary antibody (NGS in PBS alone); (**D**) corresponding p53 stained section showed 60% of cells p53 positive; assigned a score of +++ nuclear staining. (**E**) p53 shows strong nuclear staining in skin SCC. (**G**) Corresponding area of the same tumour showing moderate nuclear and weak cytoplasmic Bag-1 staining. (**F** and **H**) Higher-power images of the boxed areas in images **E** and **G** highlight the presence of predominantly nuclear p53 and Bag-1 staining, respectively. Images **A**, **E** and **G** were photographed using a × 10 objective (scale bar shown in **A**), images **B**, **D**, **F** and **H** were photographed using a × 40 objective (scale bar shown in **B**) and image **C** was photographed using a × 20 objective (scale bar shown). (**I**) Association between percentage positivity for Bag-1 and p53 in epidermal SCCs. Statistical analysis was performed using a Spearman's *ρ*-test.

As it has been reported that p53 gain-of-function mutants upregulate Bag-1 expression (Yang *et al*, 1999), 48 of the SCC sections and 9 sections of normal epidermis were stained for p53. Normal epidermis was predominantly negative for p53, as p53 is rapidly degraded in undamaged cells, but occasional p53-positive cells were seen (1–3%), indicating a damage response (Figures 6A and B). *p53* has been shown to be mutated in 50–90% of human skin SCC cases (Boukamp, 2005). High levels of p53 are indicative of p53 protein stabilisation and often indicate presence of a stable mutant form of p53 in epidermal SCC, although not all *p53* mutations are detected as protein by immunohistochemistry (Brosh and Rotter, 2009). Of the tumours, 87% showed more extensive nuclear p53-positive staining than normal epidermis (e.g., Figure 6D). Interestingly, nuclear p53 staining intensity was associated with nuclear Bag-1 staining intensity (*P*=0.042) and the percentage of cells positive for p53 also correlated with the percentage of cells positive for Bag-1 (*P*=0.003; correlation coefficient=0.431; Figure 6 – compare panels E and F with G and H, respectively; Figure 6I). However, there was no association between p53 staining and either cytoplasmic Bag-1 staining intensity or Hsp70 staining.

### Bag-1 and Hsp70 expression in epidermal dysplasia and SCC cell lines compared with normal epidermal keratinocytes

**Figure 7:**
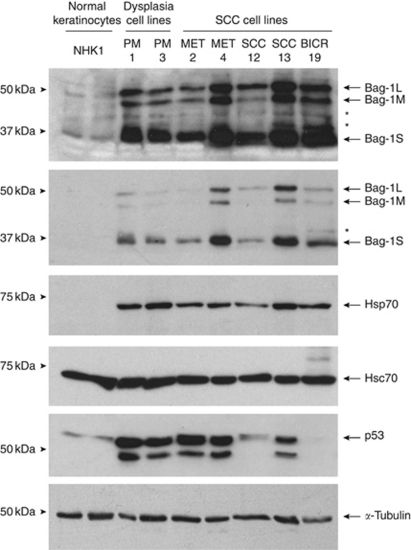
Bag-1 expression in normal epidermal keratinocytes, epidermal dysplasia and SCC cell lines. Western blot comparing Bag-1, Hsp70, Hsc70 and p53 expression of normal human epidermal keratinocytes (NHK1; passage 3; two independent sample preparations from the same patient are shown) with two epidermal dysplasia cell lines (PM1 and PM3) and five epidermal SCC cell lines (MET2, MET4, SCC-12, SCC-13 and BICR19). All cell lines were grown on the same culture medium for at least 3 days before harvest (see Materials and methods). *α*-Tubulin was used as a loading control. Asterisks (^*^) indicate non-specific antibody bands.

To ascertain whether high levels of Bag-1 might confer apoptosis resistance in poorly differentiated SCC, with potential relevance to treatment, an *in vitro* model was required. Two human epidermal dysplasia cell lines (PM1 and PM3) and five epidermal SCC cell lines (MET2, MET4, SCC-12, SCC-13 and BICR19) were assessed for Bag-1 protein expression compared with normal epidermal keratinocytes. Of these cell lines MET2, SCC-12, SCC-13 and BICR19 have known *p53* mutation and western blotting for p53 (Figure 7) showed the expected levels of p53 – SCC-12 and SCC-13 have higher levels of p53 than normal keratinocytes, and BICR19 does not express the mutant allele and has barely detectable p53 (Burns *et al*, 1993). MET2 has high levels of p53 consistent with mutation and similar high levels of p53 were detected in PM1, PM3 and MET4, perhaps indicating mutation outside of exons 5–8 that were sequenced previously (Popp *et al*, 2000) or expansion of p53 mutant clones *in vitro*. As Bag-1 expression was elevated in a subset of tumours, the hypothesis was that at least some of the SCC cell lines would express higher levels of Bag-1 than the normal epidermal keratinocytes and dysplasia cell lines. All of the cell lines expressed high levels of Bag-1 compared with normal epidermal keratinocytes (Figure 7), and three SCC cell lines (MET4, SCC-13 and BICR19) showed higher levels of all three Bag-1 protein isoforms than the PM1 and PM3 dysplasia cell lines. The predominant isoform was Bag-1S for all of the cell lines. Although normal keratinocytes showed little or no Hsp70 protein (consistent with observations *in vivo*), all of the epidermal dysplasia and carcinoma cell lines showed increased Hsp70 expression. By contrast, Hsc70 was expressed at the same levels in all of the cell lines and the normal keratinocytes.

### Bag-1 overexpression in epidermal SCC contributes to enhanced cell growth and apoptosis resistance

**Figure 8:**
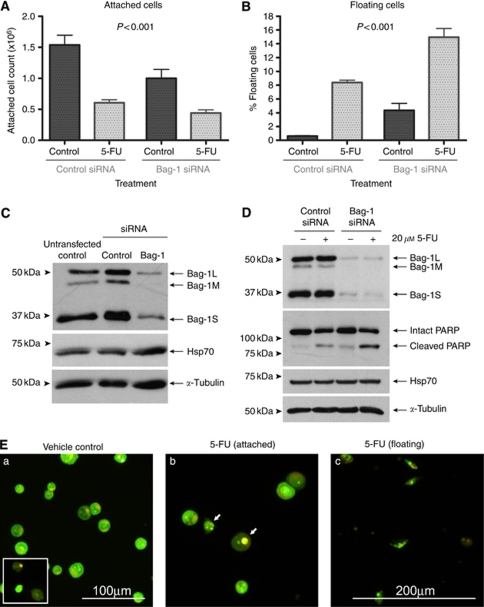
Bag-1 knockdown in the epidermal SCC cell line SCC-13 decreases cell yield and sensitises to 5-FU-induced apoptosis. Attached (**A**) and floating (**B**) cell counts from SCC-13 cells transfected with 10 nM of an irrelevant Silencer control siRNA, or Bag-1 small interfering RNA (siRNA) (a mixture of three Bag-1 siRNA sequences). Cells were treated 48 h after transfection with vehicle control (DMSO) or 20 *μ*M 5-FU. Cell counts were performed at 48 h post treatment. Data shown are the mean values obtained from three independent experiments, each performed in triplicate. Error bars represent standard error of the mean. Statistical analysis of attached cell yields and percentage of cells floating indicated a highly significant (*P*<0.001) difference between Bag-1 siRNA and Silencer control siRNA under all treatment conditions (analysis of variance and Tukey's *post hoc* test). (**C**) The Bag-1 and Hsp70 levels in SCC-13 cells transfected with 10 nM of an irrelevant control siRNA, or Bag-1 siRNA (a mixture of three Bag-1 siRNA sequences). Untransfected cells treated with oligofectamine alone were included as a control to show that the Silencer control siRNA does not significantly affect basal levels of Bag-1 or Hsp70. Note that Hsp70 levels are unaltered by Bag-1 knockdown. (**D**) Cleavage of Poly (ADP-ribose) polymerase (PARP) induced by 5-FU is enhanced subsequent to Bag-1 knockdown. The Hsp70 levels are unaffected by Bag-1 knockdown or by 5-FU treatment. Similar results were obtained in a repeat experiment. (**E**) Acridine orange/ethidium bromide staining of Bag-1 siRNA transfected SCC-13 cells treated with 5-FU or vehicle control (DMSO). (a) Attached cells treated with vehicle control (DMSO). Some occasional spontaneous apoptotic floating cells were seen (inset). (b) Attached cells treated with 5-FU. Note the presence of some apoptotic cells amongst the attached cell population in Bag-1 siRNA-transfected SCC-13 (arrows). (c) Floating cells from 5-FU-treated SCC-13. The majority of cells show classical early apoptotic morphology (condensed chromatin, cell shrinkage, maintenance of membrane potential; i.e., cells do not take up ethidium bromide and hence appear green). Images a and b were photographed using a × 20 objective (scale bar shown in a). Image c was photographed using a × 10 objective (scale bar shown in c). The images have been cropped and enlarged to more clearly show the floating cells present.

The SCC-13 cell line was selected in which to determine the consequences of Bag-1 knockdown on sensitivity to 5-FU. The SCC-13 cells were transfected with 10 nM of Silencer control siRNA, or Bag-1 siRNA (a mixture of three Bag-1 siRNA sequences), which markedly reduced levels of all three Bag-1 isoforms (Figures 8C and D). Compared with untransfected controls, the Silencer control siRNA itself did not affect Bag-1 levels (Figure 8C), nor did it affect the cell yield or apoptotic response to 5-FU (data not shown). At 48 h after siRNA transfection, cultures were treated with vehicle control (DMSO) or 20 *μ*M 5-FU for 48 h. Bag-1 knockdown alone reduced the adherent cell yield (Figure 8A) and increased cell shedding into the culture medium (Figure 8B). The Bag-1-knockdown cells were more sensitive to 5-FU treatment than Silencer control-treated cells, demonstrated by a significant (*P*<0.001) increase in cell shedding (Figure 8B). The extent of apoptotic cell shedding in the combination treatment of Bag-1 knockdown and 5-FU exposure was significantly greater than the sum of the two independent treatments (Paired *t*-test, *P*=0.004). Samples were prepared from pooled attached and floating cells from each treatment condition for western blotting analysis. Western blotting for PARP was performed as a measure of apoptotic cell death. There was an increase in the ratio of cleaved to full-length PARP in 5-FU-treated Bag-1-knockdown cells compared with 5-FU-treated Silencer control-treated cells. Examination of the cells by acridine orange and ethidium bromide staining confirmed that the shed cells were apoptotic (Figure 8E).

## Discussion

To our knowledge, this is the first study of Bag-1 expression in human epidermal SCC *in vivo* and the first demonstration that, in epidermal SCC cells in which Bag-1 is overexpressed, Bag-1 may contribute to SCC tumour proliferation as well as apoptosis resistance. Bag-1 expression is deregulated in epidermal SCC compared with normal epidermis. In our study, Bag-1 in the normal epidermis is both cytoplasmic and nuclear, as previously shown by Takayama *et al* (1998), but the predominant pattern is of nuclear staining, particularly in the spinous layer. Reduced nuclear staining intensity compared with normal epithelium is a feature of most tumours, whereas a subset showed high levels of Bag-1 throughout the cells, particularly noticeable as strong cytoplasmic staining. These findings are similar to those previously obtained in our immunohistochemical study of oral SCC (Hague *et al*, 2002). In oral SCC, reduced nuclear staining is the predominant pattern in primary tumours, but lymph node metastases tend to exhibit strong cytoplasmic staining. Shindoh *et al* (2000) reported that small primary oral tumours (T1 and T2) that had associated lymph node metastases tended to show strong Bag-1 staining. Indeed, Xie *et al* (2004) suggested that strong Bag-1 staining may be an indicator of poor prognosis for tongue SCC, a particularly aggressive subset of oral cancer.

Xie *et al* (2004) found a positive correlation between Bag-1 and Bcl-2 expression in oral SCC, and Weber *et al* (2007) found an association with Bag-1 and Hsp70 in oral SCC. We chose in this study to address the potential association between Bag-1 and Hsp70 rather than stain for Bcl-2, because although some studies have shown an increase in Bcl-2 protein levels in a subset of epidermal SCC samples (Morales-Ducret *et al*, 1995; Hussein *et al*, 2004), the general consensus from recent research suggests that Bcl-2 is infrequently deregulated in skin SCC (reviewed by Rodust *et al*, 2009). The most frequently overexpressed anti-apoptotic protein of the Bcl-2 family in epidermal SCC reported to date is Bcl-xL (Delehedde *et al*, 1999; Vasiljevic *et al*, 2009). Although there is no evidence so far that Bag-1 binds other members of the Bcl-2 family, investigating a potential link between Bag-1 and Bcl-2 and/or Bcl-xL expression in epidermal SCC could be an interesting future direction of this research.

As Hsp70 is overexpressed in human oral SCC (Kaur *et al*, 1998), it was somewhat surprising that the majority of human epidermal SCCs appeared to be negative for Hsp70 in our study, although this could reflect the concentration of antibody used. Indeed, all of the pre-malignant and SCC-derived cell lines expressed Hsp70. This may reflect *in vitro* selection of faster growing or apoptosis-resistant cells, or it could be because of the greater sensitivity of western blotting compared with immunohistochemistry for detecting protein expression at the concentration of antibody used. Interestingly, normal human breast keratinocytes screened by western blotting also showed low to negative Hsp70 expression, which corresponds with low to negative Hsp70 expression seen in normal epidermal epithelium *in vivo*. However, some Hsp70-positive areas were detected in normal skin, corresponding with sun exposure. The Hsp70 expression in keratinocytes may therefore be related to UV exposure. As our normal keratinocytes were derived from breast tissue, the lack of sun exposure may explain this difference in Hsp70 protein expression compared with the premalignant and SCC cell lines.

The 20/60 epidermal SCC sections that showed strong Hsp70 staining highlighted a subset of tumours with moderate to strong cytoplasmic Bag-1 expression. This subset of tumours tended to be classified as exhibiting moderate to poor differentiation. Hsp70 is induced by UV light exposure and has a role in the response of the epidermis to damage (Matsuda *et al*, 2010). We observed patches of strong Hsp70 staining in epithelium adjacent to skin SCCs, with particularly strong staining observed in rete pegs. The basal layer of epidermis adjacent to the tumours frequently showed much reduced nuclear Bag-1 staining compared with normal epithelium; however, elevated Bag-1 expression was seen to co-localise with Hsp70 expression in the rete pegs (data not shown). Matsuda *et al* (2010) used an Hsp70 transgenic mouse model to illustrate that Hsp70 reduces UVB-induced production of pro-inflammatory cytokines as well as reducing the induction of direct and indirect DNA damage. Hence, Hsp70 expression may contribute to protection against tumour development. Hsp70 can also protect against apoptosis (reviewed by Jäättelä, 1999) and in the Hsp70 transgenic mouse model, reduced UVB-induced apoptosis is observed in the epidermis (Matsuda *et al*, 2010). However, it is unclear whether this reflects reduced DNA damage, or whether induction of Hsp70 might permit survival of cells carrying DNA damage. It is therefore possible that Hsp70 overexpression might delay tumour formation, but act to promote carcinogenesis at later stages. Our data suggest that further study is warranted to address the hypothesis that cytoplasmic Bag-1 and Hsp70 expression together may identify tumours that are more likely to progress and potentially metastasise, as well as acquire resistance to chemotherapy.

In the epidermal dysplasia and carcinoma cell lines, Bag-1 protein was overexpressed relative to normal keratinocytes. *In vivo*, tumours are heterogeneous for Bag-1 expression and the intensity scores reflect the majority of the tumour rather than the maximal intensity of staining. Given that Bag-1 knockdown reduces the growth and enhances apoptosis of SCC-13 cells *in vitro*, it may be that selection for growth in culture has permitted the survival and expansion of cells within the tumours with high Bag-1 expression, which may be apoptosis resistant and have higher proliferative potential. However, the high level of Bag-1 in the cell lines is not simply adaptation to cell culture, since the HaCaT immortalised, non-tumourigenic epidermal cell line has lower levels of Bag-1 than SCC-13 (data not shown). The HaCaT cell line, which has a UV signature mutation in one *p53* allele and mutation in the other (Lehman *et al*, 1993), also expresses Hsp70 (data not shown), which as already discussed, may in part be related to previous UV exposure.

In their study of oral SCC, Xie *et al* (2004) reported that Bag-1 staining scores showed a positive correlation with Ki67 proliferative index. In our study, Bag-1 knockdown in the epidermal cell line SCC-13 resulted in induction of apoptosis without 5-FU treatment. However, Bag-1 knockdown also resulted in a marked reduction of cell yield that cannot be accounted for by the induction of apoptosis alone. This suggests that at least in some epidermal SCC cells, Bag-1 functions to potentiate cell proliferation as well as apoptosis, and provides support for a potential functional role for Bag-1 in promotion of cell proliferation in SCC. Takahashi *et al* (2003) reported that transient Bag-1 knockdown in HeLa cervical carcinoma cells inhibited cell growth, and in their study this led to resistance to anti-cancer drugs, whereas others have shown that stable knockdown of Bag-1 in HeLa cells sensitised the cells to cytotoxic drug-induced apoptosis (Xiong *et al* 2003). In our study, although Bag-1 reduces cell yield in SCC-13 cells, the cells are rendered more sensitive to 5-FU-induced apoptosis.

In summary, our study showed that knockdown of Bag-1 in the SCC-13 carcinoma cell line enhanced spontaneous apoptosis and sensitised the cells to apoptosis induced by 5-FU. The extent of apoptosis induced by 5-FU in the cells with Bag-1 knockdown was significantly greater than that induced by either 5-FU alone or Bag-1 knockdown alone. Continued selection during chemotherapy *in vivo* may potentially result in outgrowth of Bag-1-overexpressing sub-populations of cells that are resistant to treatment. It would be of interest to investigate the broader implications of these findings to determine the extent of Bag-1 overexpression that confers apoptosis resistance to 5-FU, and perhaps also to alternative treatments such as radiotherapy, using other cell lines from epidermal dysplasias or carcinomas. Previous studies have highlighted the importance of the interaction of Bag-1 and Hsp70 in apoptosis resistance (Townsend *et al*, 2003b, 2004). Our study indicates co-overexpression of these two proteins in a subset of tumours and points to a hypothesis that Bag-1 induces apoptosis resistance in epidermal SCC through a cytoplasmic Hsp70-dependent mechanism, as in cardiac myocytes (Townsend *et al*, 2004). Further investigation of Bag-1 function will be important, as the Bag-1:Hsp70 complex may be an appropriate target to sensitise tumour cells to apoptosis-inducing agents and overcome resistance to chemotherapy.

## Figures and Tables

**Table 1:** Details of the skin squamous cell carcinoma patients used to investigate Bag-1 and Hsp70 expression

**Test parameter**	**Category**	**Cases**	**Total cases**
Gender	Male	41	
	Female	19	60
			
Age at diagnosis (years)	Under 60	2	
	60–70	8	
	71–80	27	
	81–90	16	
	91–100	7	60
			
Site of tumour	Face/scalp	24	
	Back/neck/shoulder	7	
	Ear	9	
	Leg	8	
	Arm/hand	8	
	Other/not recorded	4	60
			
Tumour size (T)	T1 (<20 mm)	44	
	T2 (>20 and <50 mm)	4	
	T3 (>50 mm)	0	
	Not recorded	12	60
			
Differentiation	Well	33	
	Moderate	21	
	Poor	6	60

Abbreviations: Bag-1=Bcl-2-associated athanogene-1; Hsp70=heat shock protein-70.

**Table 2:** Summary of statistical analysis of Bag-1, Hsp70 and p53 staining in epidermal SCC and normal skin

**Comparison**	**Statistical test**	***P*-value**	**Significance level**	**Correlation coefficient**
*Bag-1 staining (n*=*60)*				
Cytoplasmic Bag-1 *vs* differentiation	Kruskal–Wallis	<0.001	^***^	—
Nuclear Bag-1 *vs* differentiation	Kruskal–Wallis	0.055	NS	—
				
*Hsp70 staining (n*=*60)*				
Cytoplasmic Hsp70 *vs* differentiation	Kruskal–Wallis	0.018	^*^	—
Nuclear Hsp70 *vs* differentiation	Kruskal–Wallis	0.05	NS	—
				
*Bag-1 and Hsp70 staining (n*=*60)*				
Cytoplasmic Bag-1 *vs* cytoplasmic Hsp70	Spearman's *ρ*	<0.001	^***^	0.462
Nuclear Bag-1 *vs* nuclear Hsp70	Spearman's *ρ*	0.067	NS	0.238
				
*p53 staining (n*=*48)*				
% Bag-1 *vs* % p53 positivity	Spearman's *ρ*	0.003	^**^	0.431
p53 intensity *vs* cytoplasmic Bag-1	Kruskal–Wallis	0.47	NS	—
p53 intensity *vs* nuclear Bag-1	Kruskal–Wallis	0.042	^*^	—
				
*Normal epithelium (n*=*10) vs skin SCC (n*=*60)*				
Cytoplasmic Bag-1 staining	Mann–Whitney	0.272	NS	—
Nuclear Bag-1 staining	Mann–Whitney	<0.001	^***^	—
Cytoplasmic Hsp70 staining	Mann–Whitney	0.015	^*^	—
Nuclear Hsp70 staining	Mann–Whitney	<0.001	^***^	—

Abbreviations: Bag-1=Bcl-2-associated athanogene-1; Hsp70=heat shock protein-70; NS=nonsignificant; SCC=squamous cell carcinoma. ^*^P<0.05; ^**^P<0.01; ^***^P<0.001.
